# Therapeutic effects of orthokeratology lens combined with 0.01% atropine eye drops on juvenile myopia

**DOI:** 10.5935/0004-2749.2022-0247

**Published:** 2023-03-08

**Authors:** Liwei Xiao, Jie Lv, Xiang Zhu, Xiaoyin Sun, Wen Dong, Chao Fang

**Affiliations:** 1 Department of Ophthalmology, Nanjing Integrated Traditional Chinese and Western Medicine Hospital Affiliated with Nanjing University of Chinese Medicine, Nanjing 210014, Jiangsu Province, China

**Keywords:** Atropine, Myopia, Orthokeratologic procedures, Axial length, eye, Corneal topography, Visual acuity, Contact lenses, Atropina, Miopia, Procedimentos ortoceratológicos, Comprimento axial do olho, Topografia da córnea, Acuidade visual, Lentes de contato

## Abstract

**Purpose:**

To explore the therapeutic effects of orthokeratology lens combined with 0.01% atropine eye drops on juvenile myopia.

**Methods:**

A total of 340 patients with juvenile myopia (340 eyes) treated from 2018 to December 2020 were divided into the control group (170 cases with 170 eyes, orthokeratology lens) and observation group (170 cases with 170 eyes, orthokeratology lens combined with 0.01% atropine eye drops). The best-corrected distance visual acuity, best-corrected near visual acuity, diopter, axial length, amplitude of accommodation, bright pupil diameter, dark pupil diameter, tear-film lipid layer thickness, and tear break-up time were measured before treatment and after 1 year of treatment. The incidence of adverse reactions was observed.

**Results:**

Compared with the values before treatment, the spherical equivalent degree was significantly improved by 0.22 (0.06, 0.55) D and 0.40 (0.15, 0.72) D in the observation and control groups after the treatment, respectively (p<0.01). After the treatment, the axial length was significantly increased by (0.15 ± 0.12) mm and (0.24 ± 0.11) mm in the observation and control groups, respectively, (p<0.01). After the treatment, the amplitude of accommodation significantly declined in the observation group and was lower than that in the control group, whereas both bright and dark pupil diameters significantly increase and were larger than those in the control group (p<0.01). After the treatment, the tear-film lipid layer thickness and tear break-up time significantly declined in the two groups (p<0.01).

**Conclusions:**

Orthokeratology lens combined with 0.01% atropine eye drops can synergistically enhance the control effect on juvenile myopia with high safety.

## Abstract (pt)

**Objetivo:**

Explorar os efeitos terapêuticos das lentes de ortoceratologia combinados com colírio atropina 0,01% em miopia juvenil.

**Métodos:**

Um total de 340 pacientes com miopia juvenil (340 olhos) tratados entre 2018 e Dezembro de 2020 foram divididos em Grupo Controle (170 casos com 170 olhos, lentes de ortoceratologia) e Grupo Observação (170 casos com 170 olhos, lentes de ortoceratologia combinadas com colírio atropina 0,01%). A acuidade visual melhor corrigida para longe, acuidade visual melhor corrigida para perto, dioptria, comprimento axial, amplitude de acomodação, diâmetro da pupila brilhante, diâmetro da pupila escura, espessura da camada lipídica da película lacrimal e tempo de ruptura do rasgo foram medidos antes do tratamento e 1 ano depois. A incidência de reações adversas foi observada.

**Resultados:**

Antes do tratamento, o grau esférico equivalente foi significativamente melhorado em 0,22 (0,06, 0,55) D e 0,40 (0,15, 0,72) D respectivamente no Grupo Observação e no Grupo Controle após o tratamento (p<0,01). Após tratamento, o comprimento axial foi significativamente aumentado em (0,15 ± 0,12) mm e (0,24 ± 0,11) mm respectivamente nos Grupos Observação e controle (p<0,01), enquanto, no grupo de observação, a amplitude de acomodação diminuiu significativamente e foi inferior a do Grupo Controle, e o diâmetro da pupila brilhante e o diâmetro da pupila escura aumentaram significativamente e foram maiores do que os do Grupo Controle (p<0,01). A espessura da camada lipídica da película lacrimal e o tempo de ruptura do rasgo diminuíram significativamente nos dois grupos (p<0,01) após o tratamento.

**Conclusões:**

As lentes de ortoceratologia combinadas com colírio atropina 0,01% podem melhorar significativamente o efeito controle em miopia juvenil com elevada segurança.

## INTRODUCTION

As a common refractive problem, myopia is also one of the major causes of vision disorders, and given the development of technology and changes in lifestyle, it has become a public health problem globally, seriously threa-tening the physical and mental health of adolescents^(1)^. According to a recent epidemiological survey of myopia among primary and secondary school students in China, the incidence rate of myopia reaches 55.7%, significantly higher than that in Western countries^(2)^. High myopia is a risk factor for retinal detachment, glaucoma, macular degeneration, and other eye-blinding diseases, and such a risk is augmented with the increasing severity of myopia. Therefore, effective interventions should be adopted to delay the progression of myopia and reduce the risk of these eye-blinding diseases^(3)^.

Currently, the progression of myopia is controlled primarily by optical interventions and drugs in the clinic. Orthokeratology lens, an optical intervention, is an inverse geometry-designed special corneal contact lens, and wearing it at night can flatten the central corneal curvature, reversibly reduce the degree of myopia, enhance patients’ better daytime uncorrected visual acuity, and effectively lower the axial length growth rate by 43%-63% by reducing the peripheral defocus^(4)^.

As an M-receptor antagonist, atropine may control myopia through the antimuscarinic receptors in the retina, choroid, and sclera. Moreover, 0.01% atropine was found to effectively halt the progression of myopia and reduce side effects such as paralysis of accommodation caused by high-concentration atropine and the rebound effect after drug withdrawal^(5)^. Clinically, the myopia control effect of the above-mentioned optical interventions or drugs alone is still unsatisfactory in some patients. For more effective myopia control methods, whether there is a synergistic effect between the two interventions and their safety should be examined. A few studies have demonstrated that the combination of orthokeratology lens and atropine can enhance the myopia control effect. However, the safety and efficacy of the combined treatment remain to be validated because of differences in individuals, study populations, drug concentrations, and study designs^(6-8)^.

In this study, patients with juvenile myopia were treated by orthokeratology lens combined with 0.01% atropine. The therapeutic effect and safety of this combined intervention were analyzed to obtain data supporting the selection of treatment methods.

## METHODS

This study was approved by the ethics committee of the *Nanjing Integrated Traditional Chinese and Western Medicine Hospital Affiliated with Nanjing University of Chinese Medicine*. All patients and their guardians were informed of the study’s purpose and precautions, and they provided written informed consent before enrollment. A total of 340 patients with juvenile myopia (340 eyes) treated for the first time in our hospital from 2018 to December 2020 were enrolled. The data from their right eyes were collected for analysis. All patients were from Nanjing, Jiangsu Province.

The inclusion criteria were as follows: patients aged 8-14 years; those with a spherical equivalent degree of -1.00 to -6.00 D, a degree of astigmatism with the rule of <-2.00 D, a difference in binocular spherical equivalent degree of <1.00 D, intraocular pressure of 10-21 mmHg, central corneal thickness of >0.45 mm, and corneal curvature of 39.00-46.00 D; those who had normal results in the routine ophthalmological examination; those without other eye diseases; and those whose degree of myopia increased by >0.50 D in the past year.

The exclusion criteria were as follows: patients with allergy or contraindications to atropine; those with systemic diseases or autoimmune diseases; those with contraindications to orthokeratology lenses such as dry eye, keratitis, or keratoconus; those with a history of wearing contact lens, using atropine, or eye surgery; and those with a poor compliance or unable to revisit on time.

According to the treatment methods, the participants were divided into the control group (170 cases with 170 eyes, orthokeratology lens) and the observation group (170 cases with 170 eyes, orthokeratology lens combined with 0.01% atropine eye drops).

### Treatment methods

Before fitting the orthokeratology lens, both groups underwent routine ophthalmological examination and cycloplegic optometry. The control group was treated with ALPHA orthokeratology lenses. The trial lens was selected according to the flat K and E values of the corneal topography. After tearing was stable, dynamic and static assessments of the lenses were performed under the slit lamp, and according to the lens position, activity, and fluorescence staining, the trial lens was adjusted until satisfactory fitting, with the following parameters: centralized positioning of lens, blink-induced vertical motion of the lens of 1.0-1.5 mm, 3-4 mm of flat contact area in the center, 1-2 mm of 360° fluorescence-filled area in the reverse curve area, 360° parallel contact between the positioning arc and the cornea, and 0.5 mm-wide peripheral arc fluorescent ring. The prescription was obtained through optometry on the lens. The patients were instructed to wear the lens at night for 6-8 h and take it off in the morning. They were followed up at 1 day, 1 week, 1 month, 3 months, 6 months, and 1 year after wearing the lenses. In the observation group, the orthokeratology lens combined with 0.01% atropine eye drops was used. The methods and requirements for wearing orthokeratology lenses were the same as those in the control group. Specifically, 0.01% atropine eye drops (1 mL:0.5 mg atropine sulfate injection and 1% sodium hyaluronate eye drops diluted in proportion and prepared by our hospital strictly under aseptic conditions) were given 30 min before the orthokeratology lens was worn every night, 1 drop per day, and the lacrimal sac was pressed for 10 min after each drop. For both groups, the treatment lasted for 1 year, during which the orthokeratology lenses were not replaced.

### Observation indices

After 1 year of treatment, the visual acuity, diopter, axial length, amplitude of monocular accommodation, bright/dark pupil diameters, tear-film lipid layer thickness (LLT), and tear break-up time (BUT) were examined, and the incidence of adverse events during treatment was observed.

- Visual acuity: The best-corrected distance visual acuity (BCDVA) was measured at 5 m using a standard logarithmic visual acuity chart, and the best-corrected near visual acuity (BCNVA) was measured at 40 cm using a standard logarithmic near visual acuity chart. Before measurement, a complete refractive correction was conducted, and the results were converted into LogMAR visual acuity.
- Diopter: Tropicamide eye drops (Santen Pharmaceutical, Japan) were instilled twice to dilate the pupils, with an interval of 20 min. Pupil dilation was observed 20 min after the second instillation. If there was a pupillary response to light, eye drop instillation was repeated until the response disappeared. Optometry was conducted with Topcon KR-8900 Autorefractor Keratometer (Singapore).
- Axial length: After ocular surface anesthesia, A-ultra-sound imaging was performed five times, and the average was taken. AL-Scan Optical Biometer (NIDEK, Japan) was used. The axial length was measured by detecting the signal generated by the partial superposition of light waves emitted by a light-emitting diode, with a wavelength of 830 nm.
- Pupil diameter: The bright pupil diameter was measured by a Pentacam anterior segment analyzer under the same lighting conditions, and the dark pupil diameter was measured by corneal topography in the same dark room. The measurement was repeated three times, and the average value was taken.
- Amplitude of monocular accommodation: This was measured using the push-up method. After complete refractive correction, the patient was instructed to look at a single optotype above the best visual acuity line in the near visual acuity chart, and the optotype was slowly moved closer to the patient until it became continuously blurred. The reciprocal of the plane distance between the optotype card and the glass indicated the amplitude of accommodation. The measurement was repeated three times, and the average value was taken.
- LLT: This was measured using a LipiView ocular surface interferometer. The patient was instructed to sit in front of the instrument, with the mandible was fixed on the mandibular support and the forehead stuck close to the forehead support. The position of the mandible was adjusted so that the lateral canthus was parallel to the horizontal line. During the examination, the patient was instructed to look at the indicator light in the instrument, with both eyes and blink naturally, and the LLT was recorded for 20 s in the tear-film interferogram.
- BUT: The patient’s upper palpebral conjunctiva was gently touched with a moistened fluorescein paper strip, and the patient was instructed to blink 3-4 times to evenly distribute the fluorescein in the cornea. Thereafter, examination under the slit lamp-based cobalt blue light was performed. Immediately when the patient opened his/her eyes, the time when the first dark spot appeared on the cornea was recorded as BUT.
- Adverse reactions: Adverse reactions (corneal spot-like staining, conjunctivitis, vision disorder, eye redness and itching, burning sensation, blurred vision, photophobia, etc.) during treatment were observed in both groups, and they were graded using the Efron grading scale.

### Statistical analysis

IBM SPSS Statistics for Windows version 26.0 (IBM Corp., Armonk, NY, USA) was used for statistical analysis. Measurement data were first subjected to the Kolmo-gorov-Smirnov test of normality. Except for the diffe-rences in spherical equivalent degree, the amplitude of monocular accommodation, bright pupil diameter, dark pupil diameter, and LLT before and after treatment, data followed a normal distribution and expressed as mean ± standard deviation. The differences between two groups were compared by the independent-samples *t*-test, and the paired-samples *t*-test was conducted to analyze changes before and after treatment. Non-normally distributed data were expressed as median (interquartile range) and compared between two groups by the Mann-Whitney U test. Numerical data were expressed as rate and compared by the chi-square test or Fisher’s exact test between two groups. P<0.05 was considered statistically significant.

## RESULTS

### General data of patients

Baseline data such as age, sex composition, pretreat-ment diopter, and axial length were not significantly different between the two groups (p>0.05), and they were comparable (Table 1).

**Table 1:** General data of the patients

Index	Control Group (170 cases/eyes)	Observation Group (170 cases/eyes)	p-value
Sex			>0.05
Male	70	72	
Female	100	98	
Age (Y)	10.78 ± 1.23	10.81 ± 1.34	>0.05
Spherical equivalent degree (D)	-2.96 ± 1.13	-2.98 ± 1.09	>0.05
Diopter (D)	-0.51 ± 0.07	-0.52 ± 0.08	>0.05
Axial length (mm)	25.03 ± 1.23	25.04 ± 1.19	>0.05

General data of the two groups were not significantly different.

### Visual acuity before and after treatment

Before treatment and after 1 year of treatment, the BCDVA and BCNVA were not significantly different between the two groups (p>0.05). The BCDVA and BCNVA were not significantly different in both groups after 1 year of treatment compared with those before treatment (p>0.05) (Table 2).

**Table 2:** BCDVA and BCNVA before and after treatment (LogMAR)

Index	Control Group (170 cases/eyes)	Observation Group (170 cases/eyes)	p-value
BCDVA			
Before treatment	-0.06 ± 0.06	-0.06 ± 0.05	>0.05
After 1 year of treatment	-0.04 ± 0.05	-0.05 ± 0.05	>0.05
BCNVA			
Before treatment	0.01 ± 0.02	0.01 ± 0.02	>0.05
After 1 year of treatment	0.01 ± 0.02	0.01 ± 0.02	>0.05

BCDVA and BCNVA were not significantly different between the two groups or before and after treatment. BCDNA= best-corrected distance visual acuity; BCNVA= best-corrected near visual acuity.

### Spherical equivalent degrees before and after treatment

Before treatment and after 1 year of treatment, no sig-nificant difference in spherical equivalent degree was found between the two groups (p>0.05). The spherical equivalent degree significantly increased in the two groups after 1 year of treatment compared with that before treat-ment (p<0.01), and it improved less significantly in the observation group [0.22 (0.06, 0.55) D] than in the control group [0.40 (0.15, 0.72) D] (Z=-4.435, p<0.001) (Table 3).

**Table 3:** Spherical equivalent degrees before and after treatment (D)

Index	Control Group (170 cases/eyes)	Observation Group (170 cases/eyes)
Before treatment	-3.19 ± 1.06	-3.21 ± 1.05
After 1 year of treatment	-3.64 ± 1.12^*^	-3.49 ± 1.15^*^

Compared with that before treatment, the spherical equivalent degree significantly increased in the two groups after 1 year of treatment, and it improved less significantly in the observation group than in the control group.

* P<0.05 *vs*. the same group before treatment.

### Axial lengths before and after treatment

Before treatment and after 1 year of treatment, no significant difference in the axial length was found between the two groups (p>0.05). The axial length significantly increased in the two groups after 1 year of treatment compared with that before treatment (p<0.01), and the degree of increase was significantly lower in the observation group [(0.15 ± 0.12) mm] than in the control group [(0.24 ± 0.11) mm] (t=4.182, p=0.004) (Table 4).

**Table 4:** Axial lengths before and after treatment (D)

Index	Control Group (170 cases/eyes)	Observation Group (170 cases/eyes)
Before treatment	25.02 ± 1.12	25.04 ± 1.14
After 1 of year treatment	25.24 ± 1.15^*^	25.25 ± 1.11^*^

Compared with that before treatment, the axial length significantly increased in the two groups after 1 year of treatment, and the degree of increase was significantly lower in the observation group than in the control group.

* P<0.05 *vs*. the same group before treatment.

### Amplitude of accommodation and pupil diameters before and after treatment

Before treatment, the amplitude of accommodation was not significantly different between the two groups (p>0.05). The amplitude of accommodation did not change greatly in the control group after 1 year of treatment compared with that before treatment (p>0.05), but it significantly declined in the observation group (p<0.01) and was significantly lower than that in the control group (p<0.01). After 1 year of treatment, the amplitude of accommodation varied by 0 (0, 0.25) D and -2.02 (-2.50, -1.56) D in the control and observation groups, respectively (Z=-11.254, p<0.001) (Table 5).

**Table 5:** Amplitude of accommodation before and after treatment (D)

Index	Control Group (170 cases/eyes)	Observation Group (170 cases/eyes)
Before treatment	13.07 ± 1.02	13.11 ± 1.12
After 1 year of treatment	13.13 ± 1.12	11.24 ± 1.13^*^,#

The amplitude of accommodation did not change greatly in the control group after 1 year of treatment compared with that before treatment, but it significantly declined in the observation group and was significantly lower than that in the control group.

* P<0.05 *vs*. the same group before treatment, #P<0.05 *vs*. control group.

Before treatment, the bright and dark pupil diameters were not significantly different between the two groups (p>0.05). The bright and dark pupil diameters were not significantly different in the control group after 1 year of treatment compared with those before treatment (p>0.05), but they significantly increase in the observation group (p<0.01). After 1 year of treatment, the bright and dark pupil diameters varied by 0.03 (-0.03, 0.11) mm and 0.03 (0.02, 0.10) mm and 0.82 (0.32, 1.19) mm and 0.81(0.32, 1.34) mm, respectively, in the control group and the observation group (Z=-8.082 and -8.432, p<0.001) (Table 6).

**Table 6:** Pupil diameters before and after treatment (mm)

Index	Control Group (170 cases/eyes)	Observation Group (170 cases/eyes)
Bright pupil diameter		
Before treatment	3.22 ± 0.48	3.24 ± 0.45
After 1 year of treatment	3.24 ± 0.45	4.19 ± 0.52^*^,#
Dark pupil diameter		
Before treatment	5.59 ± 0.62	5.62 ± 0.59
After 1 year of treatment	5.61 ± 0.62	6.61 ± 0.61^*^,#

Compared with those before treatment, the bright and dark pupil diameters had no significant differences in the control group after 1 year of treatment, but they significantly increased in the observation group.

* P<0.05 *vs*. the same group before treatment, #P<0.05 *vs*. control group.

### Tear-film-related parameters

Before treatment and after 1 year of treatment, no significant differences in LLT and BUT were found between the two groups (p>0.05), and both LLT and BUT significantly declined in both groups after 1 year of treatment compared with those before treatment (p<0.01). After 1 year of treatment, LLT and BUT declined by 6.02 (3.02, 10.10) nm and (1.92 ± 1.14) s and 9.05 (5.05, 10.05) nm and (2.43 ± 1.12) s, respectively, in the control and observation groups (Z=-1.234, p=0.335, t=-1.165, p=0.367) (Table 7).

**Table 7:** Tear-film-related parameters before and after treatment

Index	Control Group (170 cases/eyes)	Observation Group (170 cases/eyes)
LLT (mm)		
Before treatment	77.15 ± 15.67	77.34 ± 16.34
After 1 year of treatment	72.34 ± 14.42^*^	68.45 ± 14.53^*^,#
BUT (s)		
Before treatment	11.23 ± 1.54	11.32 ± 1.56
After 1 year of treatment	9.45 ± 0.89^*^	8.87 ± 0.92^*^,#

LLT and BUT significantly declined in the two groups after 1 year of treatment compared with those before treatment.

* P<0.05 *vs*. the same group before treatment, #P<0.05 *vs*. control group. BUT= break-up time; LLT= lipid layer thickness.

### Incidence of adverse reactions

During treatment, no allergic conjunctivitis occurred in the two groups. During the 1-year review, grade 1 corneal spot-like staining was detected in 19 (11.18%) eyes in the control group and 22 (12.94%) eyes in the observation group; however, no significant difference was found between them (p>0.05). In addition, 3 (1.76%) eyes in the control group and 8 (4.71%) eyes in the observation group had photophobia, which all occurred in the morning and was relieved in the afternoon. The incidence rate of photophobia in the observation group was higher than that in the control group (p>0.05). Moreover, 4 (2.35%) eyes in the control group and 3 (1.76%) eyes in the observation group had near-vision difficulty, but no significant difference was found between them (p>0.05). The total incidence of adverse reactions in the observation group (19.41%) was higher than that in the control group (15.29%), but no significant difference was noted between them (p>0.05).

## DISCUSSION

Orthokeratology lens, an inverse geometry-designed special corneal contact lens, is a non-surgical physical correction method. It has a flat central optical zone and a steep paracentral zone. When orthokeratology lens is worn, the cornea is reshaped through the mechanical pressure of the lens and the liquid pressure of tears, temporarily reducing the myopia diopter, so that patients can obtain better daytime uncorrected visual acuity. Moreover, orthokeratology lens can delay the growth of the axial length and control the progression of myopia in adolescents with progressive myopia^(9)^. Current studies have shown that orthokeratology lens may mitigate the progression of myopia by reducing the hyperopic defocus of the peripheral retina and can effectively reduce the axial length growth rate by 43%-63% in children with myopia^(10)^. In this study, patients with juvenile myopia were treated by orthokeratology lens combined with 0.01% atropine. The therapeutic effect and safety of this combined intervention were analyzed to obtain data supporting the selection of treatment methods.

Atropine, an alkaloid derived from *Atropa belladonna*, is a nonselective muscarinic acetylcholine receptor antagonist. Atropine eye drops acting on the antimuscarinic receptors in the retina, choroid, and sclera may increase the choroidal thickness by regulating dopamine release and interfering with scleral remodeling in myopic eyes by regulating scleral fibroblasts. In summary, atropine dose-dependently delays myopia progression and axial length extension^(11)^. However, the myopia control effect has obvious individual differences in the clinic, and the patient’s age, initial diopter, pupil size, close work time, degree of myopia, and growth and development rate may be factors influencing the myopia control effect^(12)^.

In the present study, we found that the diopter progression and axial length growth were reduced by approximately 48% and 41%, respectively, in the observation group compared with those in the control group, suggesting that the combined treatment can synergistically enhance the myopia control effect. Kinoshita et al.^(13)^ found that the axial length was increased by (0.09 ± 0.12) mm and (0.19 ± 0.15) mm in the observation and orthokeratology lens groups, respectively, after 1 year of treatment, indicating that orthokeratology lens combined with 0.01% atropine eye drops can reduce the axial length growth by 53%, basically consistent with the results of the present study. Tan et al.^(14)^ and Ji et al.^(15)^ also argued that combined treatment could increase the myopia control effect and delay both myopia progression and axial length growth. The underlying mechanism of the synergistic effect of combined treatment may be related to atropine-induced pupil enlargement. The pupil diameter may affect the myopia control effect of orthokeratology lens, and pupil enlargement can strengthen the peripheral myopia defocus effect of orthokeratology lens, thereby enhancing the myopia control effect of orthokeratology lens. Photoperiod and eyeball growth and development are closely associated, and pupil enlargement can increase retinal illumination^(16)^. In this study, the bright and dark pupil diameters increased by 0.82 (0.32, 1.19) mm and 0.81 (0.32, 1.34) mm, respectively, in the observation group after treatment, which may enhance the myopia control effect in this group.

Orthokeratology lens can reshape the cornea through the liquid pressure of the tear-film, and it can potentially affect the tear-film quality because of its direct contact with the tear-film. In this study, the BUT values were (1.92 ± 1.14) s and (2.43 ± 1.12) s and the LLT values were 6.02 (3.02, 10.10) mm and 9.05 (5.05, 10.05) nm in the control and observation groups, respectively. The change in tear-film quality by orthokeratology lens has several causes. Long-term wearing of an orthokeratology lens can weaken the corneal sensation, reduce the blink response or increase incomplete blinking, and make the tear-film lipid layer thinner, causing rapid evaporation of tears. Moreover, the flow distribution of tears varies with the change in corneal surface morphology, thus affecting tear stability^(17)^. Orthokeratology lens influences the ocular surface of adolescents, leading to meibomian gland loss, and affects tear secretion^(18)^.

Atropine is a nonselective muscarinic acetylcholine receptor antagonist. The facial nerve parasympathetic fibers regulate the tear secretion from the lacrimal gland through the lacrimal glandular branch of the maxillary nerve. Theoretically, atropine can also affect tear secretion and tear-film stability. However, in this study, this risk was not increased by 0.01% atropine, which may be related to the lower concentration of atropine used.

Atropine dose-dependently exerts a myopia control effect, and stronger myopia control often corresponds to more obvious side effects, mainly including allergic conjunctivitis, photophobia, near-vision difficulty, and rebound after drug withdrawal, all of which can be relieved by low-concentration atropine. A previous study reported that 0.01% atropine was safe and effective for myopia control^(19)^. In the present study, the bright pupil diameter was increased by 0.82 (0.32, 1.19) mm on average, and the amplitude of accommodation was decreased by -2.02 (-2.50, -1.56) D in the observation group. Compared with the control group, the most significant adverse reactions in the observation group were photophobia and near-vision difficulty. However, no significant difference was observed between the two groups, and photophobia and near-vision difficulty were milder and were gradually relieved with treatment time, suggesting that these adverse reactions had little effect. Photophobia disappeared in some children after long-term medication, possibly because of drug tolerance and compensation. Despite the slight decline in the amplitude of accommodation in patients, sufficient residual accommodation was retained, so no obvious near-vision difficulty occurred. Corneal spot-like staining is a common problem in patients wearing orthokeratology lens. In this study, the incidence rate of corneal spot-like staining was not significantly different between the observation group and the control group. Therefore, the occurrence of corneal spot-like staining may be mainly related to the orthokeratology lens, and its risk will not be significantly increased by 0.01% atropine eye drops. The ideal myopia control should be a balance between efficacy and safety.

The findings of this study demonstrate that an orthokeratology lens combined with 0.01% atropine eye drops possesses good safety, and the risk of wearing an orthokeratology lens will not be significantly increased. In conclusion, an orthokeratology lens combined with 0.01% atropine eye drops can enhance the control effect on juvenile myopia, with good safety and tolerability. However, the study had some limitations being a single-center study with a limited sample size, so the results may be biased. Further multicenter studies with larger sample sizes are in need to verify the conclusion of this study.
